# Live-cell imaging reveals decreased cAMP in a PFE-associated c.1050-3C>G PTH1R cell model

**DOI:** 10.1007/s00109-026-02668-8

**Published:** 2026-04-20

**Authors:** K. Marnet, H. Subramanian, M. Wiesler, A. Borst, D. Liedtke, G. Pattappa, D. Docheva, V. O. Nikolaev, A. Stellzig-Eisenhauer, M. Eigenthaler, M. Herrmann

**Affiliations:** 1 IZKF Group Tissue Regeneration in Musculoskeletal Diseases, University Hospital Würzburg, Würzburg, Germany; 2 Bernhard-Heine-Center for Locomotion Research, Universität Würzburg, Würzburg, Germany; 3 Institute of Experimental Cardiovascular Research, University Medical Center Hamburg-Eppendorf, Hamburg, Germany; 4 German Center for Cardiovascular Research (DZHK), Partner Site, HamburgKielLübeck, Germany; 5 Polyclinic for Orthodontics, University Hospital Würzburg, Würzburg, Germany; 6 Institute of Clinical Genetics and Genomic Medicine, University Hospital Würzburg, Würzburg, Germany; 7 Department of Musculoskeletal Tissue Regeneration, Orthopaedic Hospital König-Ludwig-Haus, University of Würzburg, Würzburg, Germany

**Keywords:** Parathyroid hormone, Parathyroid hormone-related protein, Parathyroid hormone receptor, Cyclic adenosine monophosphate, Protein kinase A, Förster Resonance Energy Transfer

## Abstract

**Abstract:**

Primary failure of eruption (PFE) is a rare autosomal disorder provoked by heterozygous mutations in the parathyroid hormone receptor 1 (*PTH1R*) gene. PTH1R is a G protein-coupled receptor (GPCR) which regulates intracellular signaling molecules like cAMP. By using CRISPR/Cas9, the pathogenic *PTH1R* variant, c.1050-3C>G, was introduced into the periodontal ligament (PDL-hTERT) cell line to investigate molecular mechanisms in a PFE in vitro model. The PDL-hTERT immortal cell line, derived from human primary PDL cells, is a well-established model for dental diseases and expresses *PTH1R*. We performed different functional assays to compare the behavior of the PDL-hTERT WT versus *PTH1R*-mutated cells. cAMP synthesis and PKA activation were compared between different cell lines by live-cell imaging using Förster Resonance Energy Transfer (FRET)-based biosensors. Phosphorylation of VASP was measured to validate and compare the PKA activation between the cell lines. In summary, our experiments show that the mutated cell line has no major phenotypic changes, but the *PTH1R* downstream signaling cascade is impaired.

**Key messages:**

- A rare autosomal disorder linked to mutations in the PTH1R gene, which encodes a G protein-coupled receptor regulating intracellular signaling, including cAMP production.
- The pathogenic PTH1R mutation (c.1050-3C>G) was introduced into a periodontal ligament (PDL hTERT) cell line to model PFE and study molecular mechanisms in vitro.
- Functional assay revealed that while the mutated cell line displayed no major phenotypic changes, the PTH1R downstream signaling cascade, including cAMP synthesis and PKA activation, was disrupted.
- Techniques like FRET-based biosensors and VASP phosphorylation assays highlighted specific impairments in signaling pathways in cells with the PTH1R mutations

**Supplementary Information:**

The online version contains supplementary material available at 10.1007/s00109-026-02668-8.

## Introduction

Primary failure of eruption (PFE, MIM#125350) is a rare autosomal, non-syndromic disorder characterized by incomplete tooth eruption during infancy, predominantly affecting posterior teeth, accompanied by stunted growth and distortion of the alveolar process within the affected region [1–4]. This leads to an open bite as the teeth remain below the occlusion level. Malfunction of the cellular eruption mechanism is considered a potential cause, as the eruption path is clear, but the teeth do not erupt [2]. Over 40 potentially pathogenic variants have been identified, linked to genetic alterations in the parathyroid hormone receptor 1, *PTH1R* [1–3, 5]. These mutations can be found in all functional regions of PTH1R potentially leading to functional changes or truncated forms of the PTH1R. The clinical occurrence of these distinct mutations is very variable and until now no clear clinical pattern can be assigned to specific mutations. First investigations with selected mutants using in vitro cell models showed changes of the cellular localization of the mutated receptors as well as dominant negative effects on the wild type PTH1R. Initial clinical studies demonstrated PFE variants to be associated with heterozygous PTH1R [6]. However, patients with homozygous variants like the PTH1R Y134S in Eiken syndrome reveal dental symptoms of PFE [7]. In both cases, a reduced expression of PTH1R levels or a dominant negative effect of a mutant receptor on expression level or function of the wild-type receptor may be crucial in the pathogenesis of PFE and explain the clinical phenotype.

Mutations in *PTH1R* are also associated with other diseases such as Jansen’s Metaphyseal Chondrodysplasia (MIM#156400), Blomstrand’s Lethal Chondrodysplasia (MIM#215045), Enchondromatosis (MIM#166000), and osteoarthritis (MIM#165720). However, unlike these conditions, PFE is unique to the dentition [8]. The similarity to dental ankylosis complicates accurate diagnosis and may lead to misdiagnosis [9].

PTH1R is a G protein-coupled receptor consisting of 593 amino acids [10] and 7 transmembrane spanning domains [11]. The gene encoding human PTH1R is located on chromosome 3 [11] and is expressed in tissues such as bone, kidney, and mammary gland [12]. With focus on PFE, its expression in osteoblasts and renal tubule cells is crucial, as PTH1R regulates calcium homeostasis, bone remodeling [1, 11], and the development and eruption of teeth [5, 13]. Binding of ligands parathyroid hormone (PTH) and parathyroid hormone-related protein (PTHrP) causes conformational changes in the receptor homodimer, activating G protein signaling cascades involving adenylate cyclase (AC) and phospholipase C (PLC) [10, 11, 14]. The receptor then activates the G proteins (Gαs, Gαq, or β-arrestin), initiating downstream signaling that regulates transcription factors. Increased adenylate cyclase activity raises intracellular cyclic adenosine monophosphate (cAMP) levels, activating protein kinase A (PKA), which phosphorylates transcription factors like CREB to regulate PTH target genes expression [11]. Additionally, cAMP activates ion channels and two isoforms of exchange factors, Epac1/cAMP-GEFI and Epac2/RAPGEF [15]. Previous studies have shown reduced cAMP response in HEK293 cells transfected with PTH1R variants, assessing their response to PTH and PTHrP [16]. Other studies of PTH1R variants in HeLa cells link PFE-associated genetic changes to functional impairments [17]. Additionally, PTH1R signaling in Prx I^+^ progenitors, expressed in mesenchymal tissue, plays a critical role in alveolar bone formation and periodontal ligament (PDL) development during tooth eruption [18].

To avoid misdiagnoses, a better understanding of the molecular mechanisms of PTH1R and its downstream signaling is crucial. Further research, particularly into the role of PTH/PTH1R in the periodontium, is essential as it could enhance understanding of PFE and related conditions. This may lead to more targeted diagnostic and therapeutic strategies. However, current knowledge is limited due to the lack of suitable in vitro models for PFE. Based on this, we chose the PDL-hTERT cell line, which is ideal for periodontitis- and PDL-engineering-related studies. It exhibits a phenotype almost identical to primary PDL-derived cells, with similar morphology and population doubling times, despite an extended lifespan [19–21].

The aim of our study was to genetically model a disease-causing condition in the PDL-hTERT cells by introducing a clinically proven variant from PFE patients. The splice site variant c.1050-3C>G leads to a truncated protein due to a single nucleotide change and affects PTH1R function [1, 2]. This variant was introduced into PDL-hTERT cells using CRISPR/Cas9. We then performed functional assays, such as proliferation and differentiation assays, to assess differences compared to control cells. Additionally, we used live-cell imaging with FRET to measure cAMP synthesis via an Epac1 biosensor [15] and PKA activation via an A-kinase activity reporter (AKAR3) biosensor [22] to further characterize active signaling pathways.

## Results

### Development of a PDL-hTERT cell line with c.1050-3C>G PTH1R variant

**Fig. 1:**
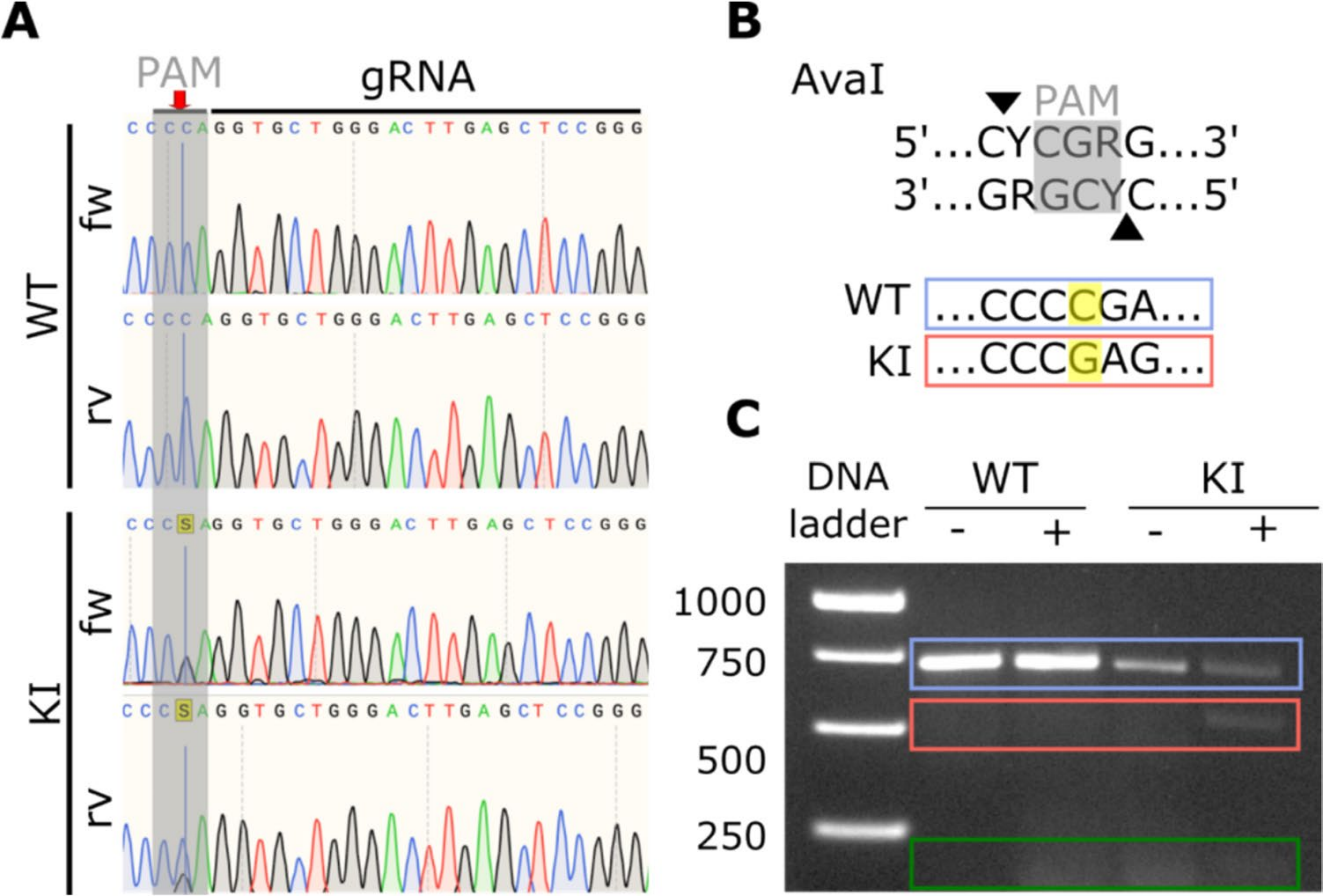
Genomic analysis of the PDL-hTERT cell line with heterozygous c.1050-3C > G *PTH1R* variant.** A** The upper two sequences show the forward (fw) and reverse (rv) wild-type gDNA sequence, while the two lower sequences display the genetically modified forward and reverse gDNA sequence. The red arrow and the blue line indicate the c.1050-3 position showing a C in the wild-type sequence and a double peak, containing a C and a G in the genetically modified sequence. The guide RNA (gRNA) as well as the PAM sequence are highlighted on top of the sequences. **B** The upper part illustrates the AvaI restriction enzyme cutting side resulting from the base exchange in general. The lower part shows the newly created AvaI restriction enzyme cutting side in the *PTH1R* sequence as it is a heterozygous mutation with only one allele showing the WT sequence (blue box) and the other allele represents the mutated allele (red box). **C** Agarose gel with a 1-kb ladder in the first column, the WT PCR product digested without (−) and with (+) AvaI in the second and third columns and the digested genetically modified PCR product without (−) and with (+) AvaI in the fourth and fifth columns. The blue box indicates the 702 bp PCR products containing the wild type sequence, while the red box shows the one digested product of the PCR with a size of 528 bp and the green box displays the other digested product of the PCR with 174 bp which indicates the exchange from C to G

We developed a clonal PDL-hTERT cell line with a heterozygous c.1050-3C>G *PTH1R* genetic variant (ClinVar database Variation ID: 13752; Accession: VCV000013752.1) using CRISPR/Cas9 and template integration. After Cas9-induced double-strand DNA breaks, the DNA template carrying the mutation was integrated at the target position in the genome. PDL-hTERT cells were transfected with Cas9 protein, a designed gRNA and the DNA template, resulting in a transfection efficiency of 11.5%. Clonal selection was performed, and Sanger sequencing confirmed the successful C to a G base change at position 1050-3 (Fig. 1A). The base exchange created a new AvaΙ restriction enzyme cutting site and genomic DNA analysis revealed a second band after digestion, confirming the mutation (Fig. 1B and C).

Following the generation of the cell line, we performed functional assays to compare key cellular properties, including proliferation rates, gene expression profiles, and signaling pathway activation. For all subsequent experiments, we utilized three different cell lines: an untransfected PDL-hTERT cell line (PDL); a gRNA/Cas9 transfected PDL-hTERT clonal cell line exhibiting the wild type PTH1R sequence (WT); and a cell line carrying the heterozygous c.1050-3C>G *PTH1R* variant (KI).

Basal gene expression analysis of genes that have been purposed as potential targets did not show any significant differences in novel generated lines (SupFig. 1).

### In vitro characterization of the cell biological effects of the heterozygous c.1050-3C>G PTH1R variant

**Fig. 2:**
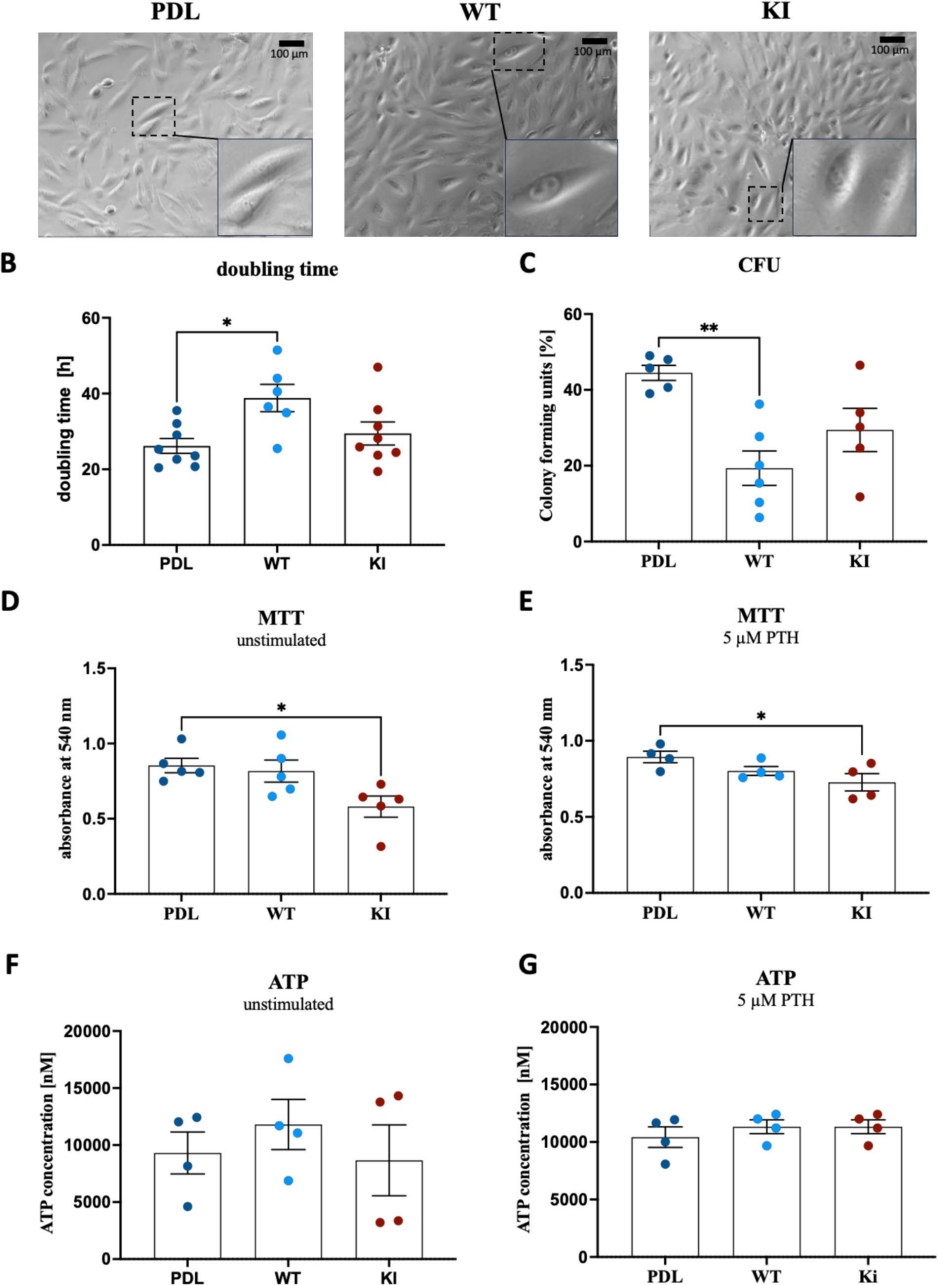
Cell morphology and growth characteristics.** A** Representative images of the three different cell lines. **B** Doubling time of the three different cell lines in hours. * for *p* = 0.0125 **C** CFU assay of the three different cell lines in %. ** for p = 0.0024 **D** MTT assay unstimulated * for *p* = 0.0214. It shows the absorbance of 540 nm. **E** MTT assay stimulated with 5 µM PTH for 24 h * for *p* = 0.0409. It shows the absorbance of 540 nm. **F** ATP assay unstimulated shows the ATP concentration in nM. **G** ATP assay stimulated with 5 µM PTH for 24 h indicates the ATP concentration in nM. Each dot represents an independent experiment. Data are presented as mean with SEM. Statistics: Ordinary one-way ANOVA followed by Dunnett’s multiple comparison test

To investigate the cell growth characteristics of the established cell lines (Fig. 2A) derived from single cells in a heterogeneous mixture, we first calculated the doubling time of the established cell lines (Fig. 2B). The results revealed that PDL cells exhibited the fastest doubling rate, with a mean doubling time of approximately 25 h. In comparison, the WT cells showed a significantly slower growth, with a doubling time of 40 h, while the KI cells had a mean doubling time of 30 h.

We performed a colony-forming unit (CFU) assay (Fig. 2C), where PDL cells formed the most colonies, followed by the KI cells, while WT cells formed significantly fewer colonies. Next, we assessed cellular metabolic activity under unstimulated conditions (Fig. 2D). PDL and WT cells exhibited similar activity, with the WT cells slightly reduced. KI cells displayed significantly lower metabolic activity than both PDL and WT cells. After PTH stimulation, PDL and WT cells showed comparable activity, but KI cells had a significantly lower response (Fig. 2E).

Intracellular ATP concentrations were also measured (Fig. 2F + G). Without stimulation, the WT cells exhibited slightly higher ATP levels (~ 11,000 nM) compared to PDL and KI cells, which had concentrations just below 10,000 nM (Fig. 2F). After 24 h of PTH stimulation, all cell lines showed similar ATP levels (10,000 nM) (Fig. 2G). No significant differences were observed in osteogenic differentiation, relative gene expression of osteogenic markers, or specific TNAP activity (CSPD assay) between the cell lines (SupFig. 2). 

**Fig. 3:**
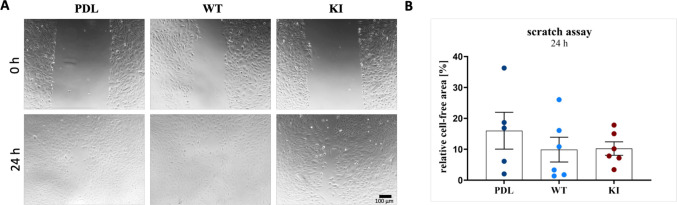
Scratch assay of the three different cell lines.** A** Representative images of Mitomycin C stimulated cell lines. The upper row is the cells directly after making the scratch, while the lower row is the cells 24 h later. **B** The graphs show the relative cell-free area in % after 24 h. Each dot represents an independent experiment. Data are presented as mean with SEM

We performed a scratch assay to compare cell migration in vitro, excluding proliferation effects using Mitomycin C (Fig. 3). Representative images at 0 h and 24 h are shown (Fig. 3A). After 24 h, PDL cells displayed the largest relative cell-free area (~ 16%), while WT and KI cells both had a smaller cell-free area (~ 10%).

### cAMP level in wild-type and mutated hTERT PDL cells after PTH stimulation

**Fig. 4:**
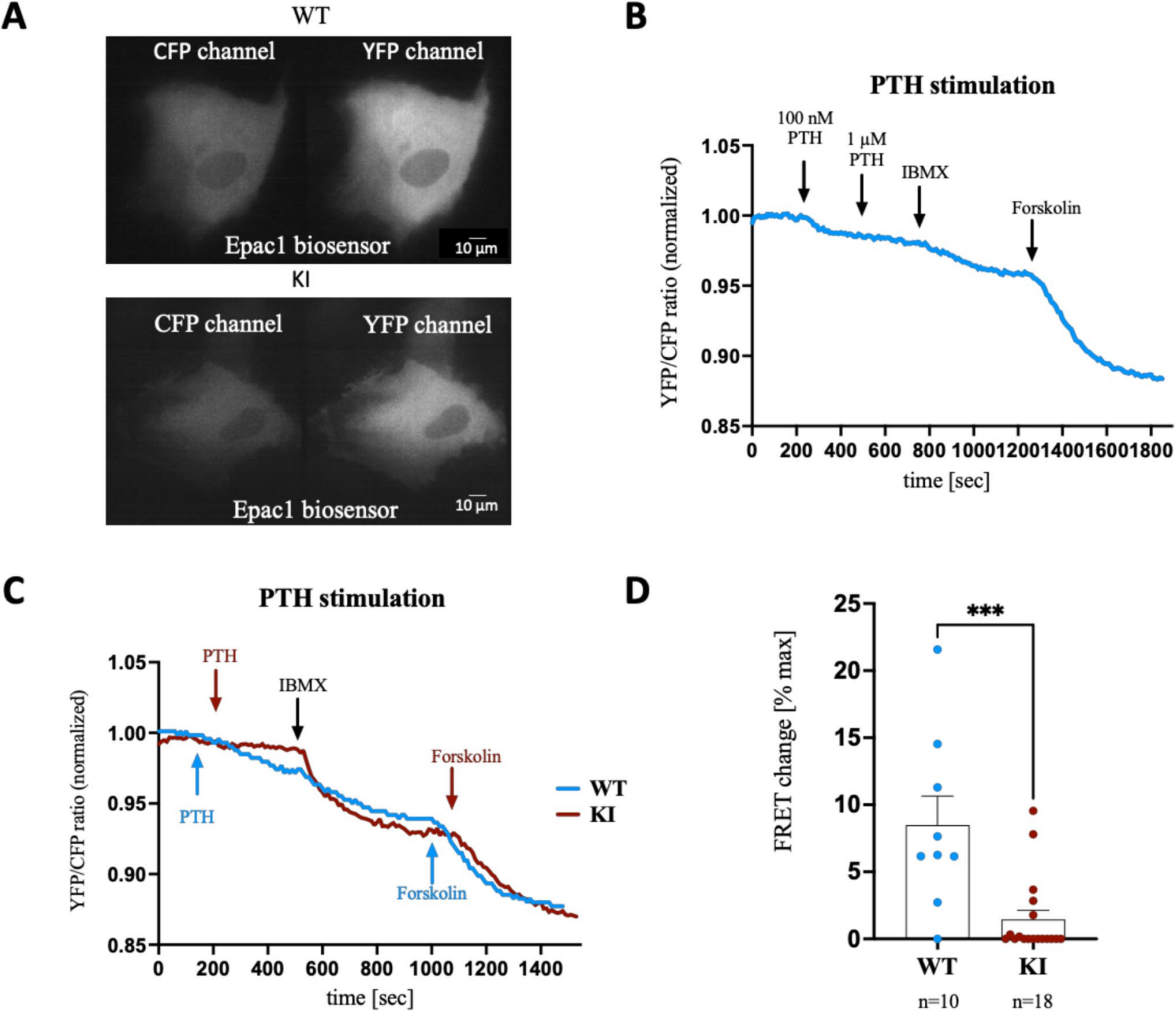
Real-time monitoring of cAMP levels in the WT and KI PDL-hTERT cells under PTH stimulation.** A** Representative CFP and YFP channel images of the wild type and mutated cells transfected with the Epac1 biosensor.** B** Representative trace of a WT cell showing the normalized FRET (YFP/CFP) ratio after PTH stimulation (100 nM and 1 µM) and the maximum cAMP increase induced by treatment with 100 µM IBMX and 10 µM forskolin. The timepoints of stimulations are indicated with the arrows. **C** Representative traces (blue: WT, red: KI) showing the normalized FRET ratio after 100 nM PTH stimulation and the maximum cAMP increase induced by treatment with 100 µM IBMX and 10 µM forskolin. **D** FRET change (% maximum cAMP) is presented (***p < 0.0006). Each dot represents an independent experiment

**Fig. 5:**
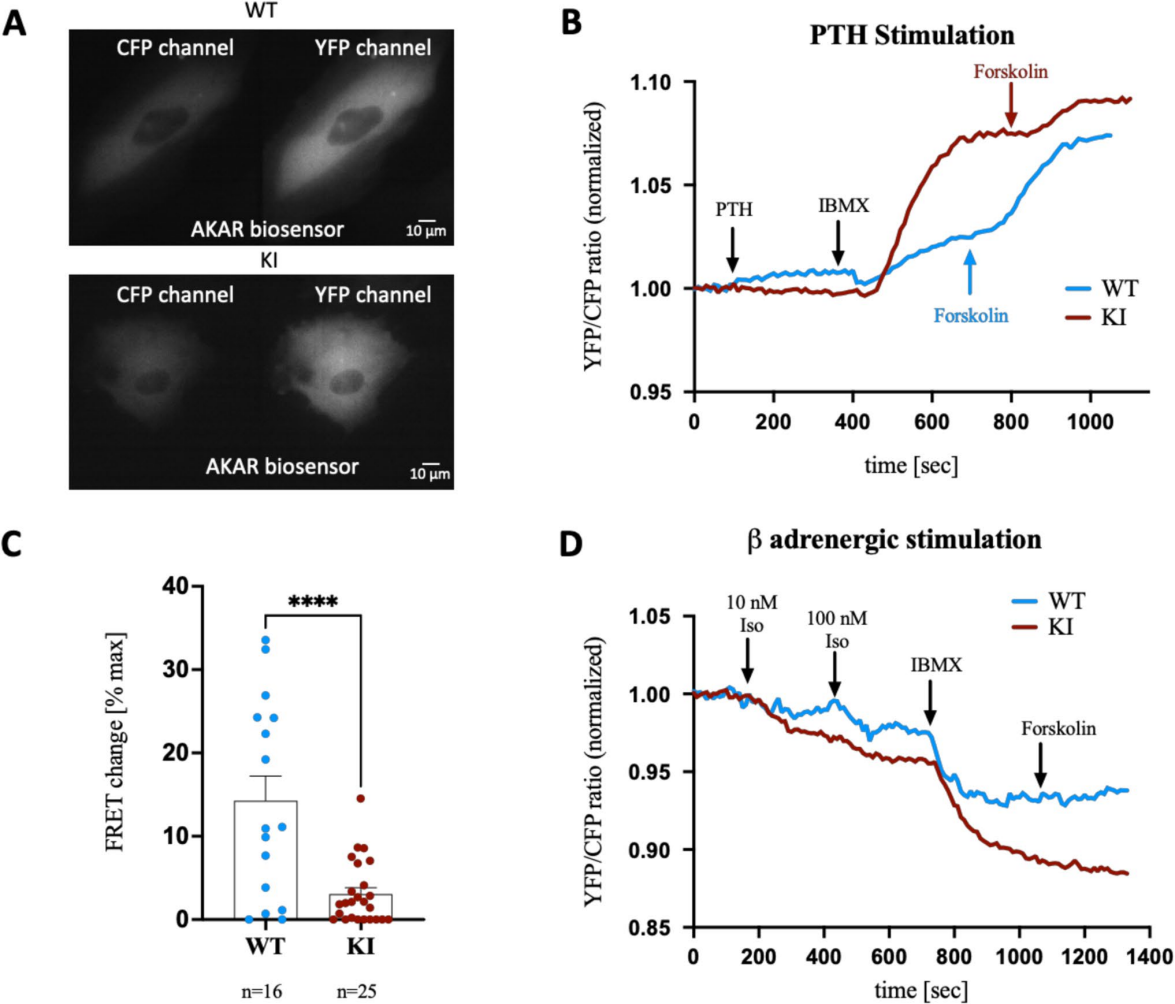
Real-time monitoring of PKA activation after PTH stimulation and of cAMP levels after β-adrenergic stimulation in wild type and mutated hTERT PDL cells.** A** Representative CFP and YFP channel images of the wild type and mutated cells transfected with the AKAR3 biosensor. **B** Representative traces (blue: WT, red: KI) showing the normalized FRET (YFP/CFP) ratio after 100 nM PTH stimulation and the maximum increase induced by treatment with 100 µM IBMX and 10 µM forskolin. **C** FRET response (% maximum) is presented (****p < 0.0001). **D** Representative traces (blue: WT, red: KI) showing the normalized FRET ratio after isoproterenol (Iso) stimulation (10 nM and 100 nM) and the maximum increase induced by treatment with 100 µM IBMX and 10 µM forskolin. Each dot represents an independent experiment

To investigate intracellular signaling after PTH1R activation, we used FRET live-cell imaging to measure cAMP synthesis and PKA activation. WT and KI cells were transfected with either the Epac1 (Fig. 4A) or the AKAR biosensor (Fig. 5A), and responses to PTH stimulation were analyzed 48 h later. YFP/CFP ratio curves quantified cAMP production during stimulation (Fig. 4B, C, 5B).

Initially, 100 nM PTH stimulation generated a clear FRET response, with minimal additional change observed at 1 µM PTH, indicating cAMP saturation at 100 nM, which was used for subsequent experiments (Fig. 4B). Sensors were saturated with maximum cAMP levels using IBMX and forskolin after PTH stimulation. WT cells exhibited significantly increased cAMP levels in response to PTH (*p* < 0.0006, FC ~ 5.8), whereas KI cells did not (Fig. 4C, D, 5B). Similar results were seen with PTHrP stimulation in the untransfected PDL-hTERT cells, but not in KI cells (SupFig. 3). 

### PKA activation in wild-type and mutated hTERT PDL cells under PTH stimulation

To assess PKA activation following PTH stimulation, WT and KI cells transfected with the FRET AKAR3 biosensor were analyzed (Fig. 5A). PKA activation was significantly higher in WT cells compared to KI cells (*p* < 0.0001, FC ~ 4.7, Fig. 5C). To underline that only PTH1R activation was impaired in KI cells, both cell lines were stimulated with isoproterenol to activate PTH-independent β-adrenergic receptors. Representative YFP/CFP traces (Fig. 5D) showed that both WT and KI cells responded to β-adrenergic stimulation by increasing cAMP level.

**Fig. 6:**
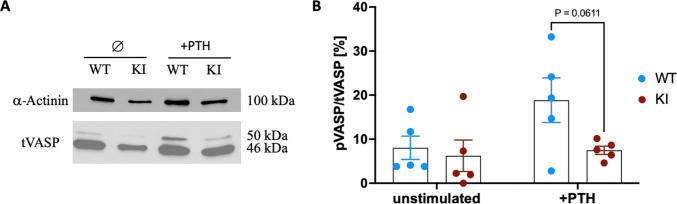
Activation of PTH1R by PTH stimulation in WT but not in KI cells.** A** Representative Western blot with unstimulated cells (∅) and stimulated cells for 10 min with 100 nM PTH (+ PTH). Blotting against Vasodilator Stimulated Phosphoprotein (tVASP) and α-Actinin was used as a loading control. **B** Quantification of phosphorylated VASP (50 kDa) relative to total VASP in %. Each dot represents an independent experiment

To validate our FRET results, we used Western blot analysis on pooled cell samples to confirm the reduced PKA response after PTH stimulation in KI compared to WT cells. We analyzed VASP (vasodilator-stimulated phosphoprotein) phosphorylation, expecting two bands: dephosphorylated at 46 kDa and phosphorylated at 50 kDa (https://www.ncbi.nlm.nih.gov/gene/7408). Both bands appeared as expected in all samples (Fig. 6A). In WT cells, the phosphorylated band (50 kDa) intensity increased after 10 min of 10 nM PTH stimulation, whereas in KI cells, the increase was minimal. Densitometric measurements of 5 blots (Fig. 6B) confirmed a strong VASP phosphorylation increase in WT cells, but only a weak increase in KI cells (*p* = 0.0611).

In summary, our FRET and Western blot results showed that PDL-hTERT KI cells harboring a c.1050-3C>G *PTH1R* variant display impaired cAMP and PKA activation after PTH stimulation. This newly established cell line serves as a valuable in vitro model to study PTH1R downstream factors and cellular mechanisms underlying PFE.

## Discussion

The *PTH1R* genetic variants linked to PFE have not been studied in disease-relevant cell types, limiting molecular insights into its pathogenesis. This study aimed to explore molecular effects within relevant cells by comparing unaltered WT cells with a potential disease-causing cell line to better understand PFE mechanisms. PDL-hTERT cells, a model for dental diseases [19, 21] expressing PTH1R, were used [2, 23]. These cells are essential for periodontal tissue formation and regeneration, acting as a source of stem cells and osteogenic progenitors with the ability to form cementum, alveolar bone, and connective tissue, essential for restoring periodontal structure and function [24–26].

Using CRISPR/Cas9, we introduced the heterozygous c.1050-3C>G splice junction mutation in the PTH1R G protein-coupled receptor, predicted to cause functional impairments such as loss of function [27]. We chose this mutation due to a distinct and consistent clinical phenotype in family members carrying this genetic change. This mutation excludes exon 12, fusing exon 11 to exon 13, resulting in a frameshift and truncated protein lacking critical transmembrane domains (TM5-7), intracellular and extracellular loops, and the cytoplasmic tail [2]. These TM domains are essential for ligand binding, signal transduction, and receptor activation [28–30]. Such changes likely lead to premature degradation or a nonfunctional receptor [2]. Notably, the heterozygous mutation leaves one allele functional, allowing potential formation of a fully functional PTH1R homodimer.

Potential off-target effects of the gene editing approach were analyzed using the CRISPOR tool [31], where no off-target binding sites for the used gRNA were predicted in silico also if up to two mismatches were allowed. This suggests that the functional changes detected within the transgenic cell lines are indeed a consequence of the heterozygous mutation within the *PTH1R* gene.

We used a CRISPR-edited WT *PTH1R* cell line as a control to ensure Cas9/sgRNA transfection did not affect cellular function. However, this WT cell line exhibited slower growth and a longer doubling time compared to both PDL and KI cells. This growth discrepancy may explain differences in functional assay results. Consistent with doubling time data, the CFU assay showed WT cells had reduced proliferation and colony-forming capacity, suggesting decreased viability. Variability in clonal properties from the non-clonal PDL-hTERT cells may account for this. Docheva et al. reported PDL-hTERT proliferation comparable to primary cells [19], indicating the *PTH1R* mutation may directly affect KI cell proliferation, as it was significantly reduced compared to PDL cells. PTH stimulation slightly increased KI metabolic activity, though it remained lower than in PDL cells. Interestingly, ATP levels equalized across all lines after PTH stimulation, suggesting intact basic cellular function. Cell migration did not correlate with doubling times. No clear differences were observed in migration capacity between the cell lines by applying an in vitro scratch assay. Migration is critical for PDL-mediated tissue regeneration [26, 32]; therefore, this should be further investigated using other assays and eventually relevant in vivo models.

Previous studies implied that the PTH receptor loses function due to the introduced mutation, promoting a detailed examination of downstream signaling. Using live-cell FRET imaging, a powerful approach [33], with Epac1 and AKAR3 biosensors, we observed that PTH stimulation increased cAMP and PKA levels in WT cells, whereas KI cells showed significantly reduced responses, confirmed by Western Blot. KI cells also exhibited decreased cAMP levels following PTHrP stimulation (supFig.3), highlighting identical roles of PTH and PTHrP in cAMP and PKA signaling [12]. The mutation’s elimination of three TMs likely impairs ligand binding and receptor activation, reducing cAMP levels and altering gene expression and metabolism [34]. β-adrenergic stimulation confirmed normal receptor function outside PTH1R, and observed cAMP degradation post-PTH stimulation suggests elevated phosphodiesterase (PDE) activity, consistent with the hypothesis from Alessandro et al. [35]. PDEs, enzymes regulating cAMP and cGMP levels, are integral to negative feedback in PKA signaling [34] and are divided into 11 families based on substrate specificity. For instance, PDEs 4, 7, and 8 target cAMP, while PDEs 5, 6, and 9 target cGMP. Others, like PDEs 1, 2, 3, 10, and 11, act on both [36]. PDE inhibitors, widely used therapeutically, may increase intracellular cAMP and enhance downstream signaling [37]. Investigating these inhibitors in PTH1R variant cell models and their potential role in dental health is a promising avenue for future research.

To note, reduced expression of *PTH1R* in KI cells would also explain the observed alterations in downstream signaling. Although gene expression analysis of *PTH1R* did not suggest any differential expression, this could not be proven on protein level due to technical reasons. Thus, lower protein expression of PTH1R in KI cells cannot be fully excluded.

In this study, we established a cell line with a clinically described disease-causing *PTH1R* variant integrated into the genome as a new in vitro model. Comparing the altered ligament cells to the healthy PDL-hTERT line allowed us to investigate and quantify signaling changes. Our findings offer key insights into the molecular mechanisms of PFE, marking an initial step toward developing innovative therapies.

## Material and methods

### Design of gRNA and HDR template for CRISPR/Cas9 genome editing

The CRISPOR website (http://crispor.gi.ucsc.edu) was utilized to design a 20-nucleotide guide RNA (gRNA) targeting the c.1050-3C>G variant (5’-GGTGCTGGGACTTGAGCTCC-3’, Merck), where the C to G nucleotide exchange lies within the PAM sequence. A 100- bp single-stranded DNA (ssDNA) template for homologous directed repair (HDR) (5’-AGTCTTAGGATGGGAACAGGAGGGATGGGAGCTAATGCCTCAACCTCCCCGAGGTGCTGGGACTTGAGCTCCGGGAACAAAAAGTGGATCATCCAGGTGCC-3’, 100 bp) was synthesized by Biomers. The template was resuspended in deionized water at 100 µM and stored at – 20 °C.

### Generation of a mutated PDL-hTERT cell line

PDL-hTERT cells [19] were cultured in DMEM (Gibco) with 10% fetal bovine serum (Capricorn) and 1% penicillin/streptomycin (Sigma) at 37 °C incubator with 5% CO_2_ and passaged weekly. Transfection used 150,000 cells, Cas9 Protein (7500 ng, Invitrogen), gRNA (50 pmol, Merck), Lipofectamine CRISPRMAX (Invitrogen), and a HDR template (200 pmol, Biomers) containing the desired mutation, following optimized Thermo Scientific’s instructions. 48 h post-transfection, cells were seeded for single cell cloning and harvested for a T7 endonuclease cleavage assay using the GeneArt™ Genomic Cleavage Detection Kit (Invitrogen). Agarose gel band intensity was analyzed with ImageJ, and transfection efficiency was calculated.

### Seeding of transfected cells for single cell expansion

200 transfected cells were seeded in a 10 cm^2^ tissue culture dish. After 24 h, single cells were marked to ensure colony origin. Individual colonies were transferred into a 24-well plate using cloning cylinders (Corning) and subsequently expanded in 6-well plates and T75 flasks to grow sufficient cells for freezing and further experiments.

### Validation of the nucleotide exchange by sequencing

Cells (1.5 × 10^6^–2.0 × 10^6^) were collected, and genomic DNA was isolated using the NucleoSpin® Tissue Kit (Macherey–Nagel). Site-specific PCR primers were designed to analyze the gRNA target (1050-3forward: 5’-GGGTCACAGGAGGCTACTTC-3’ 1050-3reverse: 5’-GTCTTTCCTGGGGTACATGGT-3’, expected PCR product size was 702 bp), and a PCR was performed with GoTaq polymerase (Promega). The annealing temperature was optimized based on primer melting temperatures.

The c.1050-3C>G substitution creates an AvaI restriction site, enabling mutation detection by digesting PCR products with AvaI (10 U/ml, New England Biolabs), and separating them on an agarose gel. WT PCR products appear as a 702 bp band, while mutant products yield additional 528 bp and 174 bp bands.

The PCR product was purified using the NucleoSpin Gel and PCR Clean up Kit (Macherey–Nagel) and sequencing PCR was performed using BigDye Terminator v1.1 (Thermo Scientific), followed by purification with the NucleoSEQ Kit (Macherey–Nagel). Sequencing results, analyzed using SnapGene and Ape software, confirmed the mutation or WT sequence.

### Cell doubling time

Equal numbers of cells (5 × 10^5^) were seeded per clone in T175 flasks. Once confluent, cells were manually counted. The population doubling time per hour was calculated using the initial seeding density, final cell counts, and culture duration. Results were confirmed in 6–8 independent experiments.

### Colony-forming unit (CFU) assay

Three hundred cells per 10 cm^2^ tissue culture dish were seeded in triplicate and cultured in standard medium. After colony formation, cells were washed with PBS, fixed, and stained with 0.5% crystal violet in methanol for 20 min at RT, then washed with sterile water. Colonies were counted, and the colony formation efficiency was calculated as the ratio of positive colonies to seeded cells. Results were confirmed in 5–6 independent experiments.

### Measurement of metabolic activity by MTT assay

Cells (2 × 10^4^) were cultured for 24 h in a 96-well plate. Cells were either directly subjected to the MTT assay or stimulated with 5 µM PTH (Sigma) for 24 h before the assay. MTT (5 mg/ml, Sigma) was added to each well (10% of the volume), and the plate was incubated for 3 h at 37 °C. Afterward, the supernatant was removed and 100 µl DMSO (Sigma) was added to dissolve the formazan crystals. The solution was mixed and incubated for 10 min at RT before measuring absorbance at 540 nm using a microplate reader (Tecan). Results were confirmed in 4–5 independent experiments.

### Cell titer glo assay

Cells (2 × 10^4^) were cultured for 24 h in a 96-well plate, with samples in triplicate and standards in duplicates. Cells were either directly subjected to the CellTiter-Glo assay or stimulated with 5 µM PTH (Sigma) for 24 h before the assay. An ATP standard curve (1 to 1000 nM) was prepared. Equal volumes of CellTiter-Glo reagent (Promega) were added to each well, and the plate was shaken for 2 min to induce lysis, followed by a 10-min incubation at RT to stabilize the luminescent signal. Luminescence was measured using a microplate reader and ATP concentrations were calculated using the standard curve. Results were confirmed in four independent experiments.

### Scratch assay

Cells (6 × 10^4^) were seeded in triplicates in a 24-well plate with 1 ml of standard medium per well. At ~ 80% confluency, cells were washed with PBS and incubated with either standard medium (control) or mitomycin C (5 µg/ml) for 30 min at 37 °C. After incubation, the cells were washed with PBS, and a scratch was introduced through the cell monolayer using a 200-µl pipette tip. Following another PBS wash, standard medium was added to each well. Images of the scratch area were captured at 0, 1, 3, and 6 h post-scratch, and image analysis was performed using Fiji with the Suarez-Arnedo plugin [38]. Results were confirmed in 3–4 independent experiments.

### Osteogenic differentiation

Cells (4 × 10^4^) were seeded and cultivated in standard medium or osteogenic medium (DMEM high glucose, 10% FCS, 10 mM β-glycerophosphate, 50 µg/ml L-ascorbic acid 2-phosphate, 100 nM dexamethasone, and 1% penicillin/streptomycin) with medium changes thrice weekly for 21 days. Osteogenic differentiation was assessed by Alizarin Red staining for mineral deposition. Cells were fixed with cold 70% ethanol, stained with 2% Alizarin Red (pH 4.2, Sigma) for 15 min, rinsed with distilled water, and imaged using a Leica (DMil) inverted microscope. The stain was eluted with 10% cetylpyridinium chloride (pH 7.0, Sigma) for 20 min at RT, and absorbance at 570 nm was measured in duplicate using a microplate reader. A standard curve (0–1000 µg/ml) quantified Alizarin Red concentration. Results were confirmed in 3–4 independent experiments.

### Quantitative real-time PCR (qPCR)

RNA was extracted from the different cell lines using the NucleoSpin RNA kit (Macherey–Nagel). One microgram of RNA was mixed with 1 µl of oligo-dT primers (Promega) and nuclease-free water. cDNA synthesis was performed by adding M-MLV Reverse Transcriptase 5× Reaction buffer, 10 mM dNTP Mix, and M-MLV reverse transcriptase enzyme (Promega). qPCR primers for osteogenic marker genes were purchased from Biomers, and 16ng of cDNA was used as the PCR template. PCR was performed with GoTag qPCR Mastermix (Promega) in a C1000 Thermal Cycler (Bio-Rad), and data was analyzed using CFX Manager (Bio-Rad). Results were confirmed in 3–5 independent experiments.

### CSPD assay

Protein from the different cell lines was extracted and lysed to evaluate specific TNAP activity. Protein concentration was calculated using the Pierce BCA Protein Assay Kit (Thermo Scientific). Samples were standardized to 10 µg using PBS/PI mixture. One half of each sample was supplemented with 1 mM levamisole (Sigma), and the other with PBS/PI. One hundred microliters of each sample, in duplicates, was transferred to a 96-well plate and an equal amount of CSPD (Merck) was added. After 5 min at 37 °C, luminescence was measured using a microplate reader.

### Förster resonance energy transfer (FRET) preparation

**Fig. 7:**
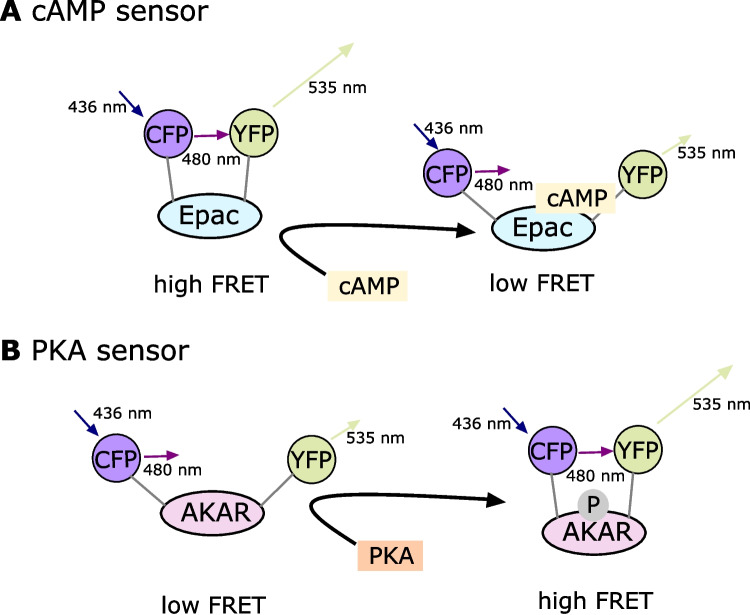
Principles of two biosensors using Epac and AKAR. **A** In the cAMP sensor, a cAMP binding domain is fused between CFP and YFP. In the presence of cAMP, the distance between CFP and YFP increases because of a conformational change. An increase in cAMP thus leads to a decrease in FRET. **B** The PKA sensor leads to an increased FRET signal with increasing cAMP concentration and subsequent PKA activation. PKA-mediated AKAR-phosphorylation leads to a conformational change that increases FRET signal

Cells were seeded onto 25-mm glass coverslips in a 6-well plate for FRET microscopy. They were transduced with an adenovirus expressing the FRET biosensor Epac1-AMP [15] or AKAR3 [22] and cultured in DMEM high glucose medium with 10% FCS and 1% penicillin/streptomycin (Fig. 7). FRET measurements were conducted 48 h after transfection.

### FRET measurements and cell imaging

Cultured cells on coverslips were placed in an Attofluor cell chamber (Thermo Scientific) with FRET buffer (144 mM NaCl, 5.4 mM KCl, 1 mM CaCl_2_, 1 mM MgCl_2_, 10 mM HEPES, pH 7.3) and positioned on a Leica DMI 3000B inverted fluorescence microscope with a 63× oil immersion objective, a pE-100 Cool LED 440-nm light source, a DV2 DualView beam splitter and an optiMOS camera (Photometrics). To minimize photobleaching, exposure time was set to 15 ms. FRET was measured as the CFP (480 nm) to YFP (535 nm) emission ratio. Images were captured using Micro-Manager software and analyzed in ImageJ.

Experiments were performed in medium containing 100 nM PTH (Sigma), 100 µM IBMX (PanReac AppliChem), or 10 µM Forskolin (Hello Bio). The cell was cropped and the CFP/YFP ratio, proportional to cAMP level, was calculated. Results were confirmed in 6–25 independent experiments.

### Western blot

Equal numbers of cells were seeded into a 6-well plate. After 48 h, cells were washed with PBS, incubated in unsupplemented medium for 2 h and stimulated for 10 min at 37 °C with FRET buffer and 100 nM PTH. Cells were then lysed with 1 × WB loading buffer, scraped, treated with 5% β-mercaptoethanol and incubated for 10 min at 95 °C. Proteins were separated by 10% SDS-PAGE and transferred onto a nitrocellulose membrane (Thermo Scientific) using standard protocols. Membranes were blocked, incubated with primary (1:3000, vasodilator stimulated phosphoprotein antibody, 0012-02, Immuno Globe) and secondary (1:2000, anti-rabbit, 7074P2, Cell signaling technology) antibodies before luminescent detection. Results were confirmed in 4–5 independent experiments.

### Quantification and statistical analysis

Data were analyzed using Prism9 (GraphPad). FRET measurements were evaluated with an unpaired *t*-test. WB data were analyzed using a two-way ANOVA, followed by Šídák’s multiple comparisons test. Functional assays were assessed with a two-way ANOVA followed by Dunnett’s multiple comparison test to determine statistical significance between groups. Normality of the datasets was tested using the D’Agostino-Pearson omnibus test, Anderson–Darling test, Shapiro–Wilk test, and the Kolmogorov–Smirnov test.

## Supplementary Information

Below is the link to the electronic supplementary material.

ESM 1 **SupFig 1: **Basal gene expression in different cell lines. The expression levels of target genes were normalized to the housekeeping gene B2M. **A** OSX. **B** RUNX2. **C** ALPL. **D** COL1A1. **E** RANKL. **F** SOST. **G** M-CSF. **H** PTH1R. Data are represented as mean with SEM (2–5) (PDF 244 KB)

ESM 2 **SupFig 2: A** Alizarin red staining with the three different cell lines after 21 days in standard (ctrl) and osteogenic differentiation (diff) medium. **B** Quantitative analysis of the Alizarin concentration in µg/ml. **C-F** Relative gene expression (qPCR) of osteogenic differentiation markers **C** OSX, **D** RUNX2 and **E** ALPL. Columns represent mean + SEM (n = 3/5). **F** CSPD assay which shows the specific TNAP activity on day 7, 14 and 21. Data represents mean + SEM (n = 3/5). (PDF 266 KB)

ESM 3 **SupFig 3: Real-time monitoring of PKA in wild type and mutated hTERT PDL cells under PTH or PTHrP stimulation. A** Representative traces (green: PDL, blue: KI) showing the normalized FRET (YFP/CFP) ratio after 100nM PTrH stimulation and the maximum increase induced by treatment with 100µM IBMX and 10µM forskolin. **B** FRET response (% maximum) in comparison for 7 wild type hTERT PDL cells and 6 KI cells. **C** Representative traces (black: WT, green: PDL) showing the normalized FRET ratio after 100nM PTH stimulation and the maximum increase induced by treatment with 100µM IBMX and 10µM forskolin. **D** FRET response (% maximum) in comparison for 6 wild type hTERT PDL cells and 10 wild type cells. (PDF 180 KB)

## Data Availability

The datasets generated during and/or analyzed during the current study are available from the corresponding author on reasonable request.
