# Supplementary material for: Live-cell imaging reveals decreased cAMP in a PFE-associated c.1050-3C>G PTH1R cell model
Source: J Mol Med (Berl). 2026 Apr 20;104(1):66. doi: 10.1007/s00109-026-02668-8 (PMC13092540; doi:10.1007/s00109-026-02668-8)
Supplement: Supplementary file 1 — SupFig 1: Basal gene expression in different cell lines. The expression levels of target genes were normalized to the housekeeping gene B2M. A OSX. B RUNX2. C ALPL. D COL1A1. E RANKL. F SOST. G M-CSF. H PTH1R. Data are represented as mean with SEM (2–5) (PDF 244 KB) [file 109_2026_2668_MOESM1_ESM.pdf]

SUPPLEMENT

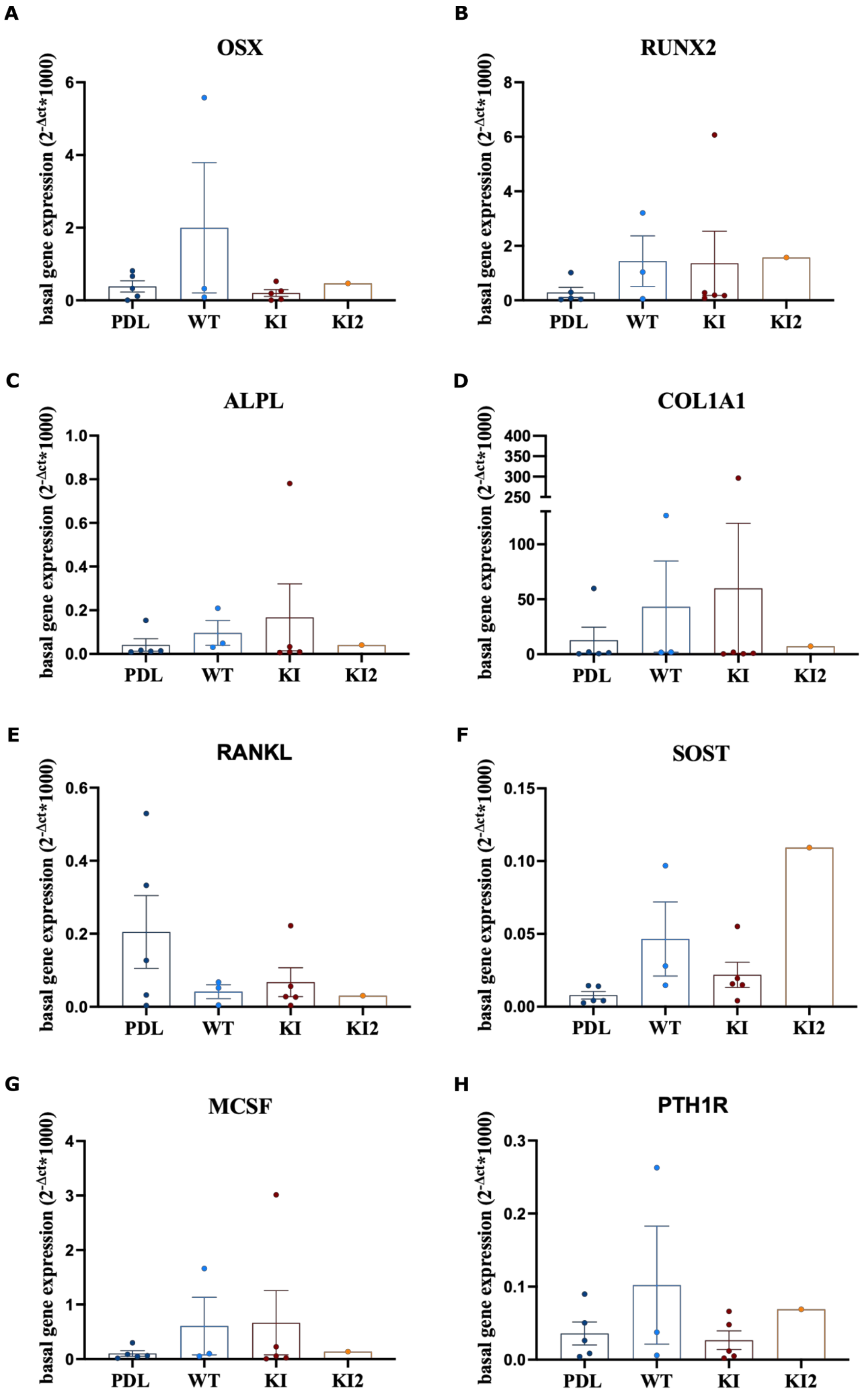

SupFig 1: Basal gene expression in different cell lines. The expression levels of target genes were normalized to the housekeeping gene B2M. **A** OSX. **B** RUNX2. **C** ALPL. **D** COL1A1. **E** RANKL. **F** SOST. **G** M-CSF. **H** PTH1R. Data are represented as mean with SEM (2-5).
