# Supplementary material for: Live-cell imaging reveals decreased cAMP in a PFE-associated c.1050-3C>G PTH1R cell model
Source: J Mol Med (Berl). 2026 Apr 20;104(1):66. doi: 10.1007/s00109-026-02668-8 (PMC13092540; doi:10.1007/s00109-026-02668-8)
Supplement: Supplementary file 2 — SupFig 2: A Alizarin red staining with the three different cell lines after 21 days in standard (ctrl) and osteogenic differentiation (diff) medium. B Quantitative analysis of the Alizarin concentration in µg/ml. C-F Relative gene expression (qPCR) of osteogenic differentiation markers C OSX, D RUNX2 and E ALPL. Columns represent mean + SEM (n = 3/5). F CSPD assay which shows the specific TNAP activity on day 7, 14 and 21. Data represents mean + SEM (n = 3/5). (PDF 266 KB) [file 109_2026_2668_MOESM2_ESM.pdf]

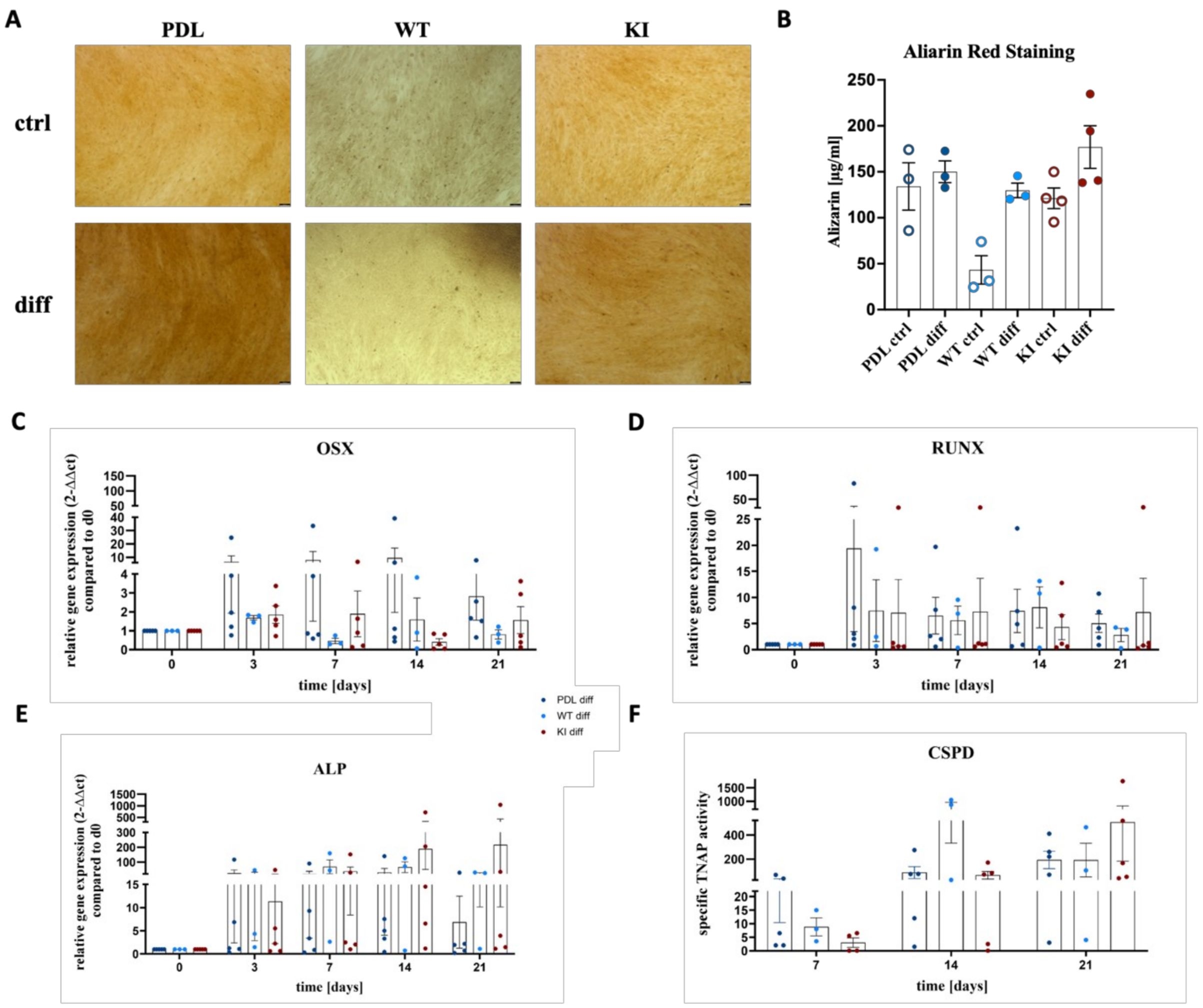

SupFig 2: A Alizarin red staining with the three different cell lines after 21 days in standard (ctrl) and osteogenic differentiation (diff) medium. **B** Quantitative analysis of the Alizarin concentration in µg/ml. **C-F** Relative gene expression (qPCR) of osteogenic differentiation markers **C** OSX, **D** RUNX2 and **E** ALPL. Columns represent mean + SEM (n=3/5). **F** CSPD assay which shows the specific TNAP activity on day 7, 14 and 21. Data represents mean + SEM (n=3/5).
