# Supplementary material for: Live-cell imaging reveals decreased cAMP in a PFE-associated c.1050-3C>G PTH1R cell model
Source: J Mol Med (Berl). 2026 Apr 20;104(1):66. doi: 10.1007/s00109-026-02668-8 (PMC13092540; doi:10.1007/s00109-026-02668-8)
Supplement: Supplementary file 3 — SupFig 3: Real-time monitoring of PKA in wild type and mutated hTERT PDL cells under PTH or PTHrP stimulation. A Representative traces (green: PDL, blue: KI) showing the normalized FRET (YFP/CFP) ratio after 100nM PTrH stimulation and the maximum increase induced by treatment with 100µM IBMX and 10µM forskolin. B FRET response (% maximum) in comparison for 7 wild type hTERT PDL cells and 6 KI cells. C Representative traces (black: WT, green: PDL) showing the normalized FRET ratio after 100nM PTH stimulation and the maximum increase induced by treatment with 100µM IBMX and 10µM forskolin. D FRET response (% maximum) in comparison for 6 wild type hTERT PDL cells and 10 wild type cells. (PDF 180 KB) [file 109_2026_2668_MOESM3_ESM.pdf]

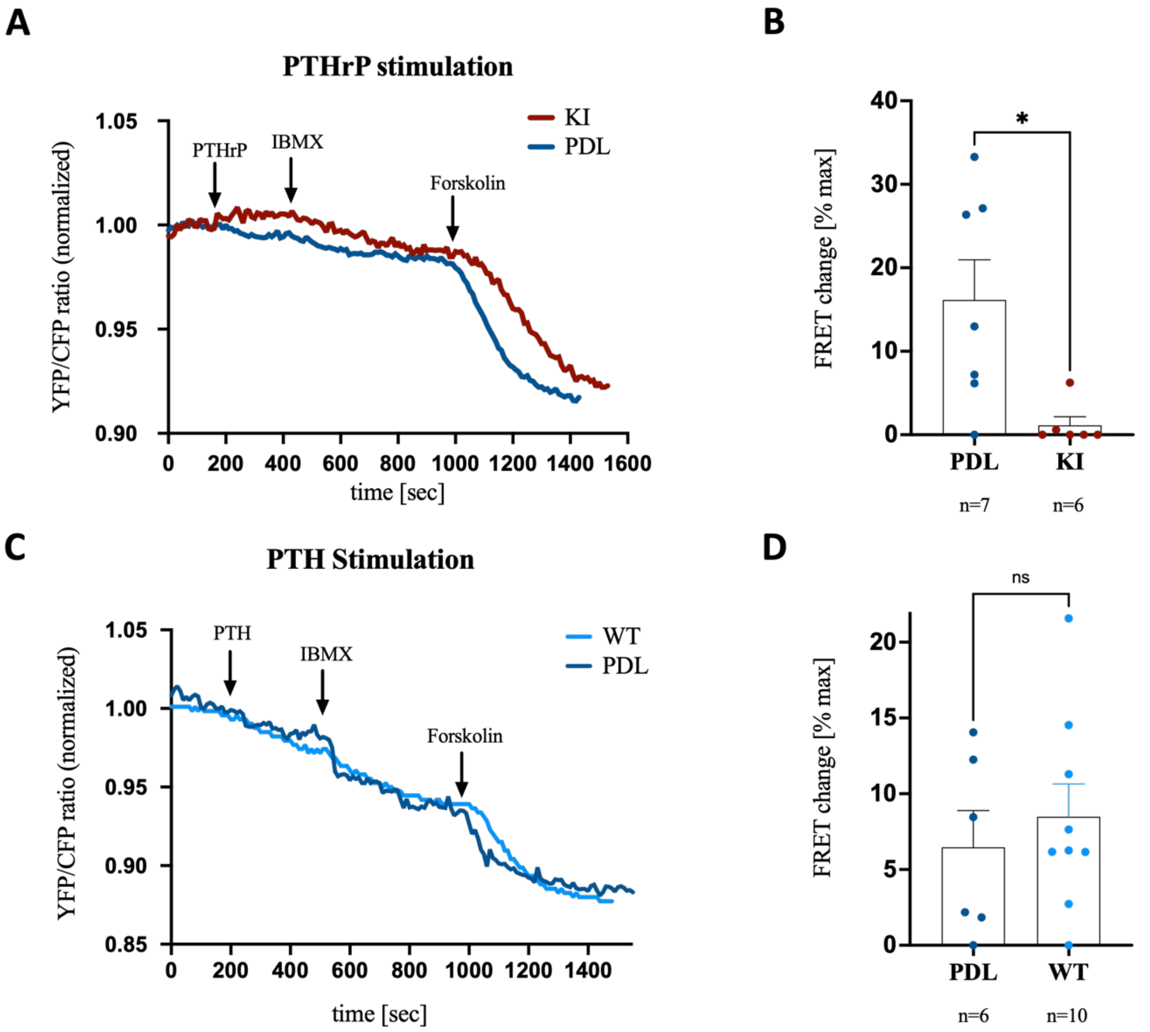

SupFig 3: Real-time monitoring of PKA in wild type and mutated hTERT PDL cells under PTH or PTHrP stimulation. A Representative traces (green: PDL, blue: KI) showing the normalized FRET (YFP/CFP) ratio after 100nM PTrH stimulation and the maximum increase induced by treatment with 100µM IBMX and 10µM forskolin. **B** FRET response (% maximum) in comparison for 7 wild type hTERT PDL cells and 6 KI cells. **C** Representative traces (black: WT, green: PDL) showing the normalized FRET ratio after 100nM PTH stimulation and the maximum increase induced by treatment with 100µM IBMX and 10µM forskolin. **D** FRET response (% maximum) in comparison for 6 wild type hTERT PDL cells and 10 wild type cells.
